# Complete deletion of *Cd39* is atheroprotective in apolipoprotein E-deficient mice[S]

**DOI:** 10.1194/jlr.M072132

**Published:** 2017-05-09

**Authors:** Marco De Giorgi, Keiichi Enjyoji, Gordon Jiang, Eva Csizmadia, Shuji Mitsuhashi, Richard J. Gumina, Ryszard T. Smolenski, Simon C. Robson

**Affiliations:** Transplant Institute and Hepatology, Department of Medicine,* Beth Israel Deaconess Medical Center and Harvard Medical School, Boston, MA; Department of Medicine,† Vanderbilt University Medical Center, Nashville, TN; Department of Biochemistry,§ Medical University of Gdansk, Gdansk, Poland

**Keywords:** atherosclerosis, cholesterol/efflux, macrophages, vascular biology, foam cells, cluster of differentiation 39, adenosine 5′-triphosphate, platelets

## Abstract

Cd39 scavenges extracellular ATP and ADP, ultimately generating adenosine, a nucleoside, which has anti-inflammatory effects in the vasculature. We have evaluated the role of Cd39 in the development of atherosclerosis in hyperlipidemic mice. *ApoE* KO (*Cd39*^+/+^/*ApoE*^−/−^) and *Cd39/ApoE* double KO (DKO) (*Cd39*^−/−^/*ApoE*^−/−^) mice were maintained on chow or Western diet for up to 20 weeks before evaluation of atherosclerotic lesions. We found that DKO mice exhibited significantly fewer atherosclerotic lesions than *ApoE* KO mice, irrespective of diet. Analyses of plaque composition revealed diminished foam cells in the fatty streaks and smaller necrotic cores in advanced lesions of DKO mice, when compared with those in *ApoE* KO mice. This atheroprotective phenotype was associated with impaired platelet reactivity to ADP in vitro and prolonged platelet survival, suggesting decreased platelet activation in vivo. Further studies with either genetic deletion or pharmacological inhibition of Cd39 in macrophages revealed increased cholesterol efflux mediated via ABCA1 to ApoA1. This phenomenon was associated with elevated plasma HDL levels in DKO mice. Our findings indicate that complete deletion of *Cd39* paradoxically attenuates development of atherosclerosis in hyperlipidemic mice. We propose that this phenotype occurs, at least in part, from diminished platelet activation, increased plasma HDL levels, and enhanced cholesterol efflux and indicates the complexity of purinergic signaling in atherosclerosis.

Atherosclerosis is a chronic inflammatory process involving large- and medium-sized arteries. This disease is characterized by the formation of atherosclerotic plaques in the vessels due to the accumulation of lipids and macrophage-derived foam cells beneath the arterial intima (1, 2). The progression of fatty streaks to more advanced lesions is driven by foam cell death, pro-inflammatory cytokine release, and the activation of other cells, in particular vascular smooth muscle cells (VSMCs) and platelets (3–5). Atherosclerosis is the leading cause of cardiovascular diseases, which account for about 40% of all deaths annually in the United States (6).

Purinergic signaling plays a key role during inflammation, fibrosis, and tissue repair (7). An increasing body of evidence suggests substantive importance of these pathways in atherosclerosis (8). Following stress, activation, or damage, cells release nucleotides in the extracellular medium, in particular ATP and ADP, which mostly act as pro-inflammatory mediators through purinergic receptor type 2 signaling (P2x_1–7_ and P2y_1,2,4,6,11–14_) (7, 8). The extracellular levels of ATP and ADP are mainly modulated by ectonucleoside triphosphate diphosphohydrolase-1 (Entpd1; also known as Cd39), which dephosphorylates ATP and ADP to AMP (9, 10). AMP is further hydrolyzed to adenosine by ecto-5′-nucleotidase (E5nt; also known as Cd73) (9, 10).

In the vasculature, Cd39 is highly expressed by the endothelium and immune cells, particularly macrophages, and to a lesser extent by VSMCs (11–13). Others and our group have demonstrated the involvement of Cd39 in the regulation of platelet activation and thrombosis (14, 15), as well as its protective role in acute inflammation (9, 12). For instance, *Cd39*-null mice have shown larger infarct sizes in a heart ischemia reperfusion injury model when compared with wt mice (16). Accordingly, the transgenic overexpression of human *CD39* conferred myocardial protection in both murine and swine ischemia reperfusion injury models (17, 18).

Recently, Kanthi et al. (19) reported that *Cd39* hemizygous mice on the *ApoE* KO background (*Cd39*^+/−^/*ApoE*^−/−^) develop more severe atherosclerotic lesions, when compared with *Cd39*^+/+^/*ApoE*^−/−^ mice. These genetically modified mice exhibited increased platelet deposition in the atherosclerotic lesions along with elevation of surrogate markers of platelet activation (19). The authors further reported that the biallelic deletion of *Cd39* on the *ApoE* KO background [double KO (DKO)] did not appear to exacerbate atherosclerosis, albeit the phenotype was heterogeneous (19). Curiously, it has been shown that the soluble CD39, or the overexpression of *CD39* in macrophages, decreases cholesterol efflux mediated by Abca1 to ApoA1 (20). These latter data suggest a potential deleterious role for Cd39 activity in foam cell formation, despite these reported benefits of the ectonucleotidase in the development of atherosclerosis.

In the present study, we further investigated these complex, potentially divergent roles of Cd39 in atherosclerosis. We note here that global deletion of *Cd39* is atheroprotective in *ApoE* KO mice, which differs from the findings in heterozygous *Cd39*^−/+^/*ApoE*^−/−^ mice reported by Kanthi et al. (19) (as also observed; not shown). The DKO *Cd39*^−/−^/*ApoE*^−/−^ mice studied here have significantly delayed progression of atherosclerosis. This protective phenotype is associated with decreased platelet activation in vitro and in vivo. Moreover, DKO mice also show higher levels of circulating HDL-cholesterol (HDL-C) along with a demonstrable increase in cholesterol efflux from macrophages, when compared with *ApoE* KO mice.

## MATERIALS AND METHODS

### Mice, genotype, and diet

*Cd39* KO mice on a C57BL/6J background were crossed with *ApoE* KO mice (C57BL/6J background) to generate *Cd39/ApoE* DKO mice. Only male mice were used in all the experimental analyses. Mice were fed with a standard chow diet or Western diet (#D12079B; Research Diets, New Brunswick, NJ) for up to 20 weeks before being euthanized for experiments. The Beth Israel Deaconess Medical Center Institutional Animal Care and Use Committee approved all animal experiments in accordance with National Institutes of Health guidelines.

### Oil Red O staining of aortas

Aortas from mice were cleaned from adventitial tissues and fat, and fixed overnight in 4% PFA. Oil Red O staining was performed as previously described (21). Briefly, aortas were opened longitudinally and stained with Oil Red O (Sigma-Aldrich, St. Louis, MO) for 40 min at 37°C. The excess of Oil Red O was removed by washing with 60% isopropanol for 5 min at 37°C. The atherosclerotic lesion size was measured by the area of positive staining divided by the total aortic surface in images digitized in ImageJ software (ver. 1.46r; National Institutes of Health; http://rsb.info.nih.gov/ij/).

### Blood lipid analysis

After fasting overnight, mice were anesthetized briefly with 5% isoflurane (Abbott Animal Health, Abbott Park, IL) inhalation and 200 μl of blood were collected by retro-orbital plexus using a noncoated hematocrit glass capillary (Chase Scientific Glass, Rockwood, TN). Blood was allowed to clot at 4°C overnight and then centrifuged at 5,000 *g* for 15 min (4°C). The obtained supernatant was centrifuged again at 11,000 *g* for 2 min (4°C). Serum total cholesterol, LDL-cholesterol (LDL-C), and HDL-C were measured using commercial colorimetric assays (Wako Cholesterol E, Wako L-type LDL-Cholesterol, Wako HDL-Cholesterol E; Wako Diagnostics, Richmond, VA) according to the manufacturer’s instructions. HDL-C levels were measured in plasma from bone marrow (BM)-transplanted mice by using HDL and LDL/VLDL quantification kit (Sigma-Aldrich) following the manufacturer’s instructions. Four volumes of blood were collected from the inferior vena cava in a tube containing 1 vol of 3.2% sodium citrate. Blood was centrifuged at 5,000 *g* for 15 min at room temperature and then the supernatant was centrifuged again at 11,000 *g* for 3 min at room temperature. Plasma was stored at −80°C until use.

### Quantification of the lesion size in the aortic sinus

The heart was perfused with PBS and fixed in 10% formalin for 48 h. The fixed hearts were dehydrated and embedded in paraffin and then cross-sectioned. The sections were discarded until the valve cups became visible. Four sections at 50 μm intervals were collected in the same slide and stained with hematoxylin and eosin for necrotic core quantification, Elastica van Gieson staining for atherosclerotic lesion area quantification, or Sirius red staining for collagen quantification.

### Hematoxylin and eosin staining

Sections were deparaffinized and rehydrated through two changes of xylene (4 min each), 100% ethanol (4 min each), 95% ethanol (2 min each), and finally one change of 70% ethanol (2 min) and then rinsed three times in distilled water. Then nuclei were stained with Gill’s Hematoxylin II (American MasterTech Scientific, Lodi, CA) for 2 min followed by 10 dips in acid alcohol and 20 s in ammonium hydroxide. Cytoplasm was stained with Eosin Y (American MasterTech Scientific) for 30 s. Sections were then dehydrated by 10 dips in 95% ethanol (twice), 30 s in 100% ethanol (twice), and ethanol was removed by two changes of xylene (2 min each) before covering the slide. Pictures were taken by using Eclipse E600 (Nikon) and SPOT software 5.1 (Spot Imaging Solutions, Sterling Heights, MI). The necrotic area, defined as the unstained acellular area inside the lesion, was blindly measured in four 50 μm distant sections per animal using ImageJ software.

### Elastica van Gieson staining

Weigert’s hematoxylin solution was prepared by mixing equal volumes of solution A (1% hematoxylin in 100% ethanol) and solution B (4 ml of 30% ferric chloride and 1 ml of concentrated HCl in 100 ml of distilled water).

Sections were deparaffinized and rehydrated as described above. Nuclei were stained with Weigert’s hematoxylin for 20 min followed by washing in water and differentiation in acid alcohol. Sections were washed in water for 5 min and then stained with van Gieson’s staining solution (90 ml of 1.6% saturated picric acid, 10 ml of 1% acid fuchsine, and 1.25 ml of glacial acetic acid) for 2 min. After washing in water, sections were dehydrated in 100% ethanol (ten dips, two times) and ethanol was removed by two changes of xylene (30 s and 2 min, respectively). Pictures were taken by using Eclipse E600 and SPOT software 5.1. The lesion area was blindly measured in four 50 μm distant sections per animal by using ImageJ software.

### Sirius red staining

Weigert’s hematoxylin solution was prepared as described above. Sections were deparaffinized and rehydrated as described above. Nuclei were stained with Weigert’s hematoxylin for 8 min and then slides were washed in water for 10 min. Collagen was stained with 1% Picro-Sirius Red (Sigma-Aldrich) for 1 h, followed by two changes of acidified water. Sections were dehydrated in three changes of 100% ethanol (30 s twice and 1 min) and then two changes of xylene (2 min each) before covering the slide. Pictures were taken by using Eclipse E600 and SPOT software 5.1. Intra-lesion collagen area was blindly measured in four 50 μm distant sections per animal by using ImageJ software.

### Immunohistochemical analysis of the aortic sinus lesions

Sections were deparaffinized and rehydrated as described above. Then they were incubated in 10 mM sodium citrate buffer (pH 6) and antigen retrieval was performed by using 2100 Antigen Retriever. Sections were rinsed in PBS and blocked with 7% horse serum (Vector Laboratories) for 30 min at room temperature. Then sections were incubated overnight at 4°C with the appropriate primary antibody diluted in PBS. The antibodies used were: rat anti-mouse Mac3 (M3/84, #550292, 1:150; BD Pharmingen); mouse anti-human Smc α actin (1A4, #M0851, 1:150; Dako); rabbit anti-human Cd3g (EPR4517, #ab134096, 1:100; Abcam); rabbit anti-mouse cleaved caspase 3 (5A1E, #9664, 1:100; Cell Signaling); and polyclonal sheep anti-mouse Cd39 (#AF4398; 1:500; R&D Systems). The day after, sections were washed twice in PBS (5 min each) and endogenous peroxidase activity was blocked by 3% H_2_O_2_ (Sigma-Aldrich) incubation for 10 min at room temperature. Sections were washed three times by PBS (5 min each) and then incubated with a secondary antibody: biotinylated rabbit anti-rat IgG (3 μl in 1 ml of PBS; Vector Laboratories); biotinylated goat anti-rabbit IgG (1.2 μl in 1 ml of PBS; Vector Laboratories); biotinylated horse anti-mouse IgG (3 μl in 1 ml of PBS; Vector Laboratories); or biotinylated donkey anti-goat IgG (1:500 in PBS; Jackson Immuno Research) for 1 h at room temperature. After two washes in PBS (5 min each), Vectastain Elite ABC kit (Vector Laboratories) was applied to sections for 30 min at room temperature and the excess of reagent was removed by two changes in PBS (5 min each). Color development was performed by applying a drop per section of ImmPACT DAB peroxidase (HRP) substrate kit (Vector Laboratories). Nuclei were counterstained with hematoxylin (Sigma-Aldrich) and dehydrated through two changes of 95% ethanol (30 s each), two changes of 100% ethanol (2 min each), and two changes of xylene (2 min each) before cover slide application. Pictures were taken by using Eclipse E600 and SPOT software 5.1. IHC quantification was blindly performed by using ImageJ software.

In some cases, Cd39 and Cd39L1 were detected by frozen immunohistochemistry. Briefly, hearts were perfused with PBS. Then they were frozen on a cryostat mount with OCT compound (Tissue Tek) and trimmed by a cryostat until the valve cups became visible. Sections were fixed in cold 7% formalin in acetone for 3 min and then washed with PBS. Sections were blocked as described above and incubated overnight at 4°C with rabbit anti-mouse Cd39 or Cd39L1 at a concentration of 1:1,000 (provided from Dr. Sevigny, Université Laval, Canada). On the next day, sections were washed twice in PBS and endogenous biotin was blocked by using Avidin/Biotin blocking kit (Vector Laboratories) following the manufacturer’s instructions. Blocking of endogenous peroxidase activity, secondary antibody incubation, and the following steps were performed as previously described for formalin-fixed paraffin-embedded sections.

### TUNEL assay

Sections were deparaffinized and rehydrated as described above. TUNEL assay was performed by using VasoTACS in situ apoptosis detection kit (Trevigen) following the manufacturer’s instructions exactly. Proteinase K solution was applied for 30 min. Color was developed for 7 min and then counterstained for 30 s. Pictures were taken by using Eclipse E600 and SPOT software 5.1. Blue positive staining was blindly quantified using ImageJ software.

### Blood lipid analysis

After fasting overnight, mice were anesthetized briefly with 5% isoflurane (Abbott Animal Health) inhalation and 200 μl of blood were collected by retro-orbital plexus using a noncoated hematocrit glass capillary (Chase Scientific Glass). Blood was allowed to clot at 4°C overnight and then centrifuged at 5,000 *g* for 15 min (4°C). The obtained supernatant was centrifuged again at 11,000 *g* for 2 min (4°C). Serum total cholesterol, LDL-C, and HDL-C were measured using commercial colorimetric assays (Wako Cholesterol E, Wako L-type LDL-Cholesterol, Wako HDL-Cholesterol E; Wako Diagnostics) according to the manufacturer’s instructions.

### Platelet aggregation and half-life analyses

Platelet-rich plasma was isolated as previously described (14). Platelets used for analyses were isolated from mice fed the chow diet for 20 weeks. Platelet count was adjusted with HEPES-Tyrode’s solution to have a final concentration of 2 × 10^5^ platelets/μl. Platelet aggregation was measured by a Lumi-aggregometer and AggroLink software (Chrono-Log, Havertown, PA). Samples of 0.25 ml were stimulated with ADP (2.5 μM) for 5 min while stirring at 37°C and light transmission was measured over time.

For the determination of platelet turnover, platelets were labeled in vivo by injecting 200 μl of 3 mg/ml NHS-biotin (Pierce; EZ-Link). Every 24 h for 4 days, 5 μl of blood were collected from the tail vein into 125 μl of HEPES/Tyrode’s solution supplemented with anticoagulants (85 mM sodium citrate, 69 mM citric acid) and 20 mg/ml glucose. Samples were incubated with FITC anti-mouse Cd41 (eBioscience) and PE-streptavidin (BD Pharmingen) for 30 min. In these studies, 50,000 events were analyzed at BD FACSCalibur flow cytometer.

### BM-derived macrophage differentiation and culture

Macrophages were differentiated from BM cells as previously described (22). Briefly, BM cells were harvested by flushing the femurs with sterile PBS and then cultured in medium (RPMI1640 containing 10% FBS and 1× penicillin/streptomycin) supplemented with 10 ng/ml of murine macrophage colony-stimulating factor (M-csf; PeproTech) for 5 days at 37°C, 5% CO_2_. Medium was replaced on the second day of differentiation. BM-derived macrophages (BMDMs) were detached by using macrophage detachment solution (5 mM EDTA and 4 mg/ml lidocaine HCl in PBS) and by gentle scraping. Macrophages were characterized by FACS staining with APC Cd11b (M1/70, #17-0112-82, 1:100; BioLegend), FITC F4/80 (BM8, #11-4801-81, 1:100; eBioscience) and PE Cd39 (5F2, #12-3390-80, 1:100; eBioscience). APC rat IgG2b (RTK4530, #400611, 1:100; BioLegend), FITC rat IgG2b (eB149/10H5, #11-4031-81, 1:100; BioLegend), and PE mouse IgG1 (P3.6.2.8.1, 1:100; eBioscience) were used as isotype controls.

### BMDM analyses

The foam cell formation assay was performed as previously described with some modifications (23). Briefly, BMDMs were seeded at 8 × 10^4^ cells/well in 24-well plates and, on the following day, human oxidized LDL (oxLDL) (25, 100, and 200 μg/ml; BT-910; Alfa Aesar) were added in complete medium supplemented with 10 ng/ml M-csf. No oxLDL was added in the untreated controls. Cells were cultured for 24 h and, after washing with PBS, fixed with 4% PFA for 30 min. Cells were washed with PBS for 1 min and rinsed with 60% isopropanol for 15 s. Then cells were stained with Oil Red O at 37°C for 5 min followed by 15 s of destaining with 60% isopropanol and three washes with PBS (3 min each). In order to quantify foam cells, Oil Red O was extracted with 100% isopropanol for 5 min with gentle shaking and the absorbance at 518 nm was measured using an Epoch microplate spectrophotometer (BioTek). One hundred percent isopropanol was used as background control. Proteins were extracted using modified RIPA solution [50 nM Tris-HCl (pH 7.4), 150 mM NaCl, 1% NP-4, 0.25% sodium deoxycholate] and quantified by DC Protein assay (Bio-Rad, Hercules, CA) following the manufacturer’s protocol. Absorbance at 750 nm was read using an Epoch microplate spectrophotometer (BioTek).

The viability assay was performed as previously described with some modifications (24, 25). BMDMs were seeded at 10^4^ cells/well in 96-well plates in medium supplemented with 10 ng/ml M-csf. The following day, cells were incubated with different concentrations of human oxLDL (25, 100, and 200 μg/ml) in medium without M-csf for 24 h. Control cells were cultured in complete medium (supplemented with 10 ng/ml M-csf) or medium without M-csf. Viability was analyzed by using Cell Counting Kit-8 (Dojindo Molecular Technologies) according to the manufacturer’s protocol. Absorbance at 450 nm was read using an Epoch microplate spectrophotometer (BioTek).

The cholesterol efflux assay was performed as previously described (26) with some modifications. BMDMs were seeded at 8 × 10^4^ cells/well in 24-well plates in medium supplemented with 10 ng/ml M-csf. Subsequently, cells were incubated with 1 μCi/ml [^3^H]cholesterol (PerkinElmer) in complete medium for 24 h. After two washes with PBS, cells were incubated for 18 h in equilibration medium (1% BSA in RPMI plus 1× penicillin/streptomycin and 10 ng/ml M-csf) supplemented with 0.3 mM 8-Br-cAMP (Sigma-Aldrich) in order to induce ABCA1 expression. After one wash with PBS, cells were incubated with 10 μg/ml ApoA1 (BT-927; Alfa Aesar) in equilibration medium. No acceptor ApoA1 was used as background efflux control. Where indicated, 25 U/ml Apyrase (from potatoes, grade VII; Sigma-Aldrich) or 26 μM POM1 (provided by Dr. Christa E. Müller, University of Bonn, Germany) were added to the medium containing ApoA1. After 8 h of incubation, supernatant was collected and centrifuged at 12,000 *g* for 10 min in order to remove any detached cells. The amount of [^3^H]cholesterol was measured with a scintillation counter. The remaining adherent cells were lysed in 1 N NaOH overnight and the cell radioactivity was measured with a scintillation counter. Efflux was expressed as the percentage of radioactivity in the supernatant over the total radioactivity (supernatant and cells). Assays were performed at least in triplicate wells.

### BM transplantation

BM cells were harvested by flushing the femurs and tibias of the donor mice with sterile PBS. After washing in PBS, cells were counted and resuspended in sterile 0.9% saline solution at a concentration of 3.0 × 10^7^ cells/ml. Mice (5 weeks old) were γ-irradiated at total 9 Gy over 10 min and then received 6 × 10^6^ BM cells from donor mice in 200 μl of 0.9% saline solution via penile vein injection. The recipient mice were allowed to recover for 4 weeks and then fed a Western diet for 12 weeks.

### Control of BM reconstitution

Eighty microliters of blood were collected by tail vein incision in a tube containing 20 μl of 3.8% sodium citrate. Erythrocytes were lysed by adding 600 μl of erythrocyte lysis solution (Qiagen, Valencia, CA) for 5 min in agitation. Then blood was centrifuged at 260 *g* for 5 min and the pellet was resuspended in 10 ml of cold PBS. Cells were centrifuged at 260 *g* for 5 min and then resuspended in 100 μl of FACS buffer (1% FBS in PBS) containing the appropriate antibodies. Cells were stained at 4°C in the dark for 1 h and then pelleted after adding 1 ml of FACS buffer. Cells were resuspended in 500 μl of FACS buffer and analyzed at BD FACSCalibur flow cytometer. B-lymphocytes were stained with APC Cd45 (30-F11, #103112, 1:100; BioLegend), FITC Cd19 (6D5, #115506, 1:100; BioLegend), and PE Cd39 (5F2, #12-3390-80, 1:100; eBioscience). APC rat IgG2b (RTK4530, #400611, 1:100; BioLegend), FITC rat IgG2a (R35-95, #554688, 1:100; BD Biosciences), and PE mouse IgG1 (P3.6.2.8.1, #12-4714-41, 1:100; eBioscience) were used as isotype controls. Thirty thousand events from lymphocyte gate were acquired in each analysis.

### ELISA for platelet factor 4

Plasma samples were isolated as described above. Platelet factor 4 (PF4) ELISA on plasma samples was performed by using RayBio Mouse PF-4 ELISA kit (RayBiotech, Norcross, GA) following the manufacturer’s instructions. Plasma samples were diluted 1:200 and added to PF4 antibody-coated plates for an overnight incubation at 4°C. Absorbance at 450 nm was measured using an Epoch microplate spectrophotometer (BioTek, Winooski, VT).

### VSMC isolation

VSMCs were isolated from murine aorta as previously described (27). In order to isolate enough cells, three to four aortas were pooled together. Briefly, aortas were dissected and the adventitial tissue and fat were removed. Then aortas were cut into 1–2 mm rings and put in digestion with Collagenase type II (Gibco) for 6 h (7.5 mg of Collagenase II in 5.5 ml of culture medium: DMEM containing 10% FBS, 1× penicillin/streptomycin, and 1% glutamine). The lysate was pelleted and the cells were resuspended in 700 μl of culture medium and cultured at 37°C, 5% CO_2_. After 5 days, the medium was replaced and, once confluent, cells were trypsinized and expanded. Cells were cultured up to the third passage. The purity of VSMCs was checked by immunofluorescence staining with anti-actin, α-Smooth Muscle-Cy3 (1A4, #C6198, 1:5,000; Sigma-Aldrich). Cd39 expression was evaluated by FACS analyzing 30,000 cells using PE Cd39 (5F2, #12-3390-80, 1:100; eBioscience) and PE mouse IgG1 (P3.6.2.8.1, #12-4714-41, 1:100; eBioscience) as isotype control.

### VSMC analyses

For the proliferation assay, VSMCs were seeded at 10^4^ cells/well in 96-well plates in complete medium. The following day, medium was replaced with starvation medium (0.2% FBS in DMEM plus 1× penicillin/streptomycin and 1% glutamine). After 24 h of starvation, cells were incubated with normal medium (10% FBS), 10 ng/ml platelet-derived growth factor (Pdgf) (PeproTech), or 100 μM ATP (Sigma-Aldrich) or both stimuli in starvation medium. Proliferation was measured at 48 h using Cell Counting Kit-8 (Dojindo Molecular Technologies) according to the manufacturer’s protocol. Absorbance at 450 nm was read using an Epoch microplate spectrophotometer (BioTek).

Migration assay was performed as previously described (28). VSMCs were starved overnight (0.2% FBS in DMEM medium with 1× penicillin/streptomycin and 1% glutamine) before performing the migration assay. An 8 μm pore size Transwell system (Costar) was used. Cells (3 × 10^4^) in 100 μl of starvation medium were seeded in the upper chamber and allowed to migrate for 10 h toward the lower chamber containing 500 μl of migration medium. Ten percent FBS, 10 ng/ml Pdgf, or 100 μM ATP diluted in starvation medium was used as chemoattractant in the lower chamber, while starvation medium alone was used as basal migration control. After 10 h, the insert was removed and washed in PBS and the cells were fixed in 4% PFA for 30 min at room temperature. After a PBS washing, cells were stained in 0.2% crystal violet for 30 min at room temperature. The excess of dye was removed by several changes in PBS. Nonmigrated cells were gently removed using a cotton swab. Pictures were taken by using Celigo (Cyntellect). Cell counting in five causal fields of view was performed by using ImageJ software.

### Statistical analyses

Statistical analyses were performed with GraphPad software (Prism). Values are expressed as mean ± SEM. Student’s two-tailed *t*-test and one-way ANOVA followed by Tukey’s post hoc test were used for comparing, respectively, two and more than two variables. Data were considered statistically significant when *P* < 0.05.

## RESULTS

### Global genetic deletion of *Cd39* protects against atherosclerosis

In order to investigate the contribution of Cd39 in atherosclerosis, we crossed *ApoE* KO to *Cd39* KO C57BL6 mice generating DKO mice. We first evaluated the expression of the two main vascular ectonucleotidases (Cd39 and Cd39L1) in the aortic sinus of *ApoE* KO and DKO mice. As expected, Cd39 was mainly expressed in the vascular endothelium, foam cells, and VSMCs of *ApoE* KO mice in a predictable manner (supplemental Fig. S1A). Cd39L1 staining was detected only in the adventitia with no differences in the expression pattern between *ApoE* KO and DKO mice (supplemental Fig. S1B).

**Fig. 1.**
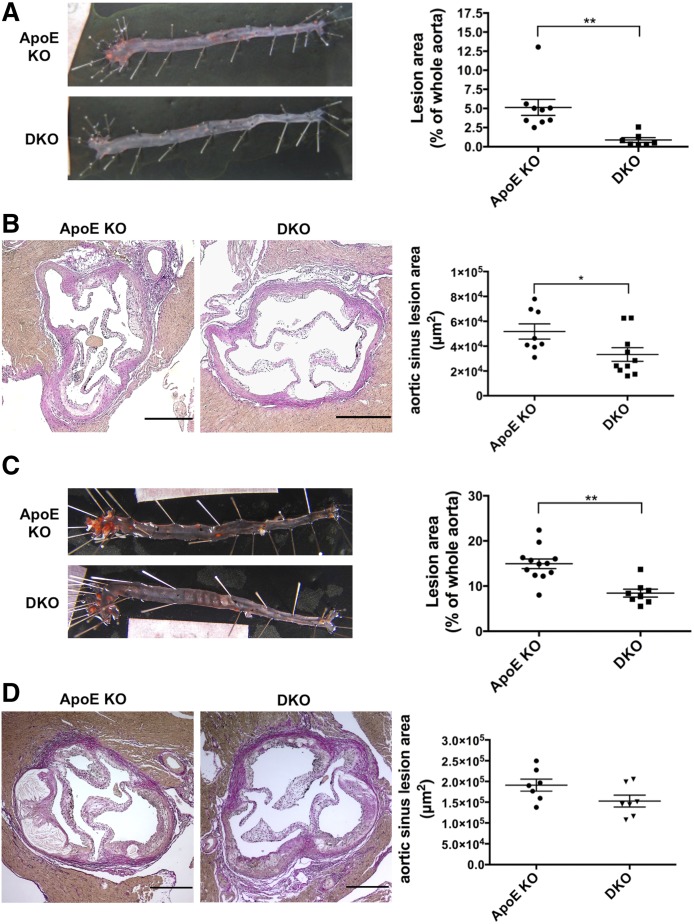
*Cd39* deletion attenuates the formation of atherosclerotic lesions in *ApoE*-deficient mice. Male *ApoE* KO and DKO mice were fed a chow diet (n = 9 and 7 mice, respectively) (A) or a Western diet (n = 12 and 8 mice, respectively) (C) for up to 20 weeks and atherosclerotic lesion sizes were determined by Oil Red O staining of the whole aorta. Atherosclerotic lesions were also evaluated by Elastica van Gieson staining of the aortic root of *ApoE* KO and DKO mice fed a chow diet (n = 8 and 10 mice, respectively) (B) or a Western Diet (n = 7 mice in both groups) (D). Intimal plaque area was measured in four 50 μm distant sections per each animal. Data are presented as mean ± SEM. Representative images are shown. Scale bar in (B) and (D) is 250 μm. *P < 0.05; **P < 0.01.

Mice were fed a chow diet for up to 20 weeks and atherosclerotic lesions were evaluated by Oil Red O staining of the entire aorta. DKO mice developed lesions of significantly decreased size, as expressed by the percentage of positive disease area and compared with *ApoE* KO mice [0.9 ± 0.3% (n = 7) vs. 5.1 ± 1.0% (n = 9); *P* < 0.01; **Fig. 1A**]. Moreover, DKO mice showed significantly smaller lesions in the aortic root than *ApoE* KO mice [3.3 × 10^4^ ± 0.5 × 10^4^ μm^2^ (n = 10) vs. 5.2 × 10^4^ ± 0.6 × 10^4^ μm^2^ (n = 8); *P* < 0.05; Fig. 1B].

To examine whether *Cd39* deletion could also protect hyperlipidemic mice, *ApoE* KO and DKO mice were fed a Western diet for up to 20 weeks. Atherosclerotic lesions in the whole aorta from DKO mice were almost 50% less compared with those from the *ApoE* KO mice [8.4 ± 0.9% (n = 8) vs. 14.9 ± 1.1% (n = 12); *P* < 0.01; Fig. 1C]. Lesions were uniformly distributed along the aorta in both groups. This difference was not so evident in the aortic root, where DKO mice showed a trend toward smaller lesions compared with *ApoE* KO mice [1.5 × 10^5^ ± 0.1 × 10^5^ μm^2^ (n = 7) vs. 1.9 × 10^5^ ± 0.1 × 10^5^ μm^2^ (n = 7); *P* = 0.08; Fig. 1D].

**Fig. 2.**
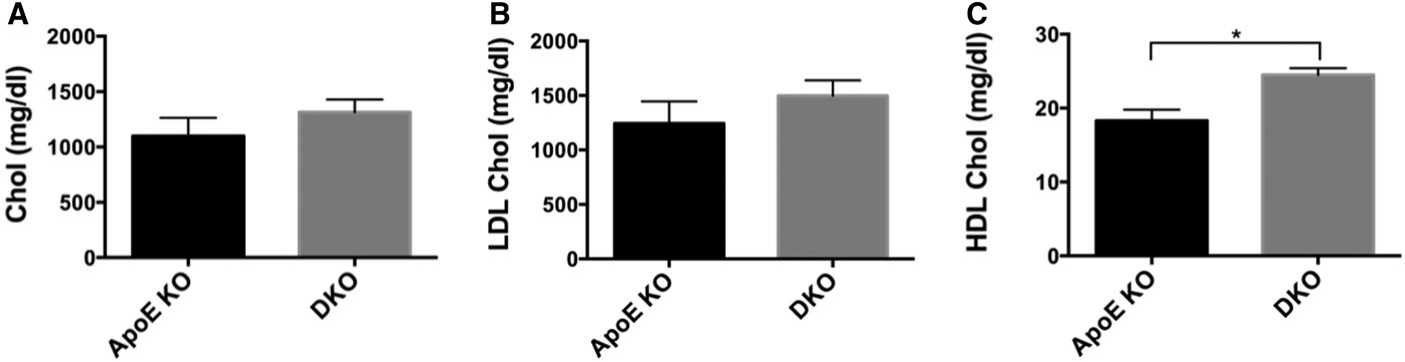
*Cd39* deletion results in higher HDL levels in serum from Western diet-fed mice. Peripheral blood from Western diet-fed *ApoE* KO and DKO mice was analyzed for levels of total cholesterol (A), LDL-C (B), and HDL-C (C) (n = 4–5 mice per group). Data are presented as mean ± SEM. **P < 0.01.

To determine whether this difference was linked to changes in circulating lipoproteins, we examined the serum lipid profiles of mice fed the Western diet. No significant differences were detected in total cholesterol and LDL-C levels between the two groups (**Fig. 2A, B**). However, DKO mice had significantly higher levels of HDL-C than *ApoE* KO mice (Fig. 2C).

Taken together, these data show that the global deletion of *Cd39* is protective against development of atherosclerosis in *ApoE*-deficient mice.

### Global deletion of *Cd39* in *ApoE*-null mice results in decreased macrophage accumulation in fatty streaks and less necrotic area in advanced vascular lesions

**Fig. 3.**
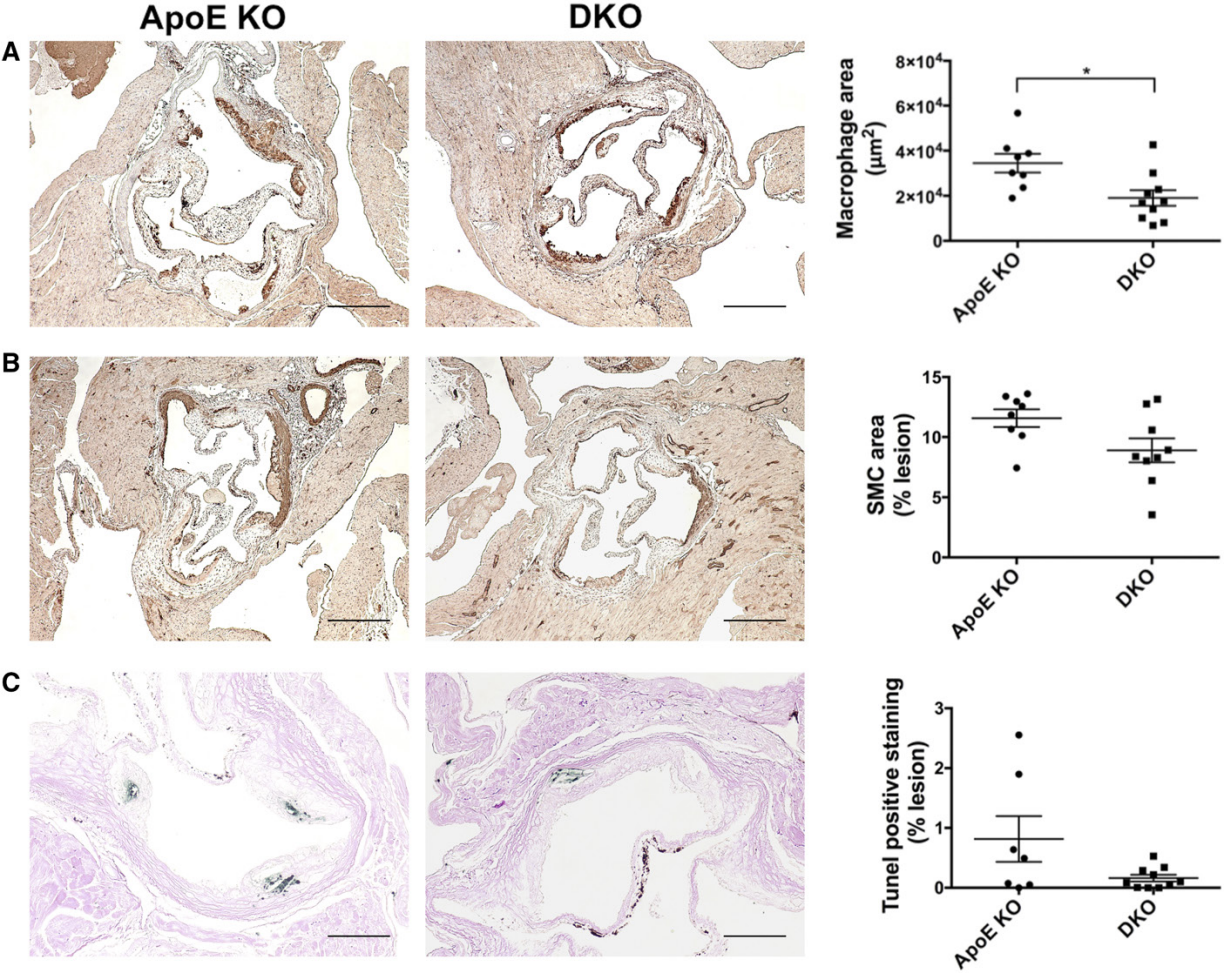
*Cd39* deletion results in decreased macrophage numbers in fatty streaks from chow diet-fed mice. A: Macrophage levels were evaluated by Mac3 immunohistochemistry on aortic sinuses (one section per animal; n = 8 *ApoE* KO and 10 DKO mice). B: VSMCs infiltration was evaluated by Smc α actin immunohistochemistry on aortic sinus (one section per animal; n = 8 *ApoE* KO and 9 DKO mice). C: Intra-lesion apoptosis was investigated by TUNEL assay on aortic sinus (one section per animal; n = 7 *ApoE* KO and 10 DKO mice). Representative images are shown (scale bar is 250 μm in (A) and (B), 100 μm in (C). Quantification data are presented as mean ± SEM of the area staining or the percentage staining of the total plaque area. Setting a color threshold in ImageJ identified positive staining. *P < 0.05.

We then analyzed the effects of *Cd39* deficiency on the composition of fatty streaks in the aortic sinus from chow diet-fed mice. At this stage, lesions were composed mostly of macrophages and very few VSMCs (**Fig. 3**). DKO mice had significantly smaller macrophage-positive areas than did the corresponding *ApoE* KO mice [19,039 ± 3,459 μm^2^ (n = 10) vs. 34,480 ± 4,153 μm^2^ (n = 8); *P* < 0.05; Fig. 3A]; whereas VSMCs infiltration was similar between the two groups [*ApoE* KO: 11.6 ± 0.7% (n = 8) vs. DKO: 8.9 ± 1.0% (n = 9); Fig. 3B]. In addition, DKO mice exhibited a trend toward decreased apoptosis of foam cells in fatty streaks [0.2 ± 0.06% (n = 10) vs. 0.8 ± 0.4% (n = 7); Fig. 3C]. T cell infiltration was minimal in both groups, as shown by the very few Cd3-positive cells inside the lesions (supplemental Fig. S2).

**Fig. 4.**
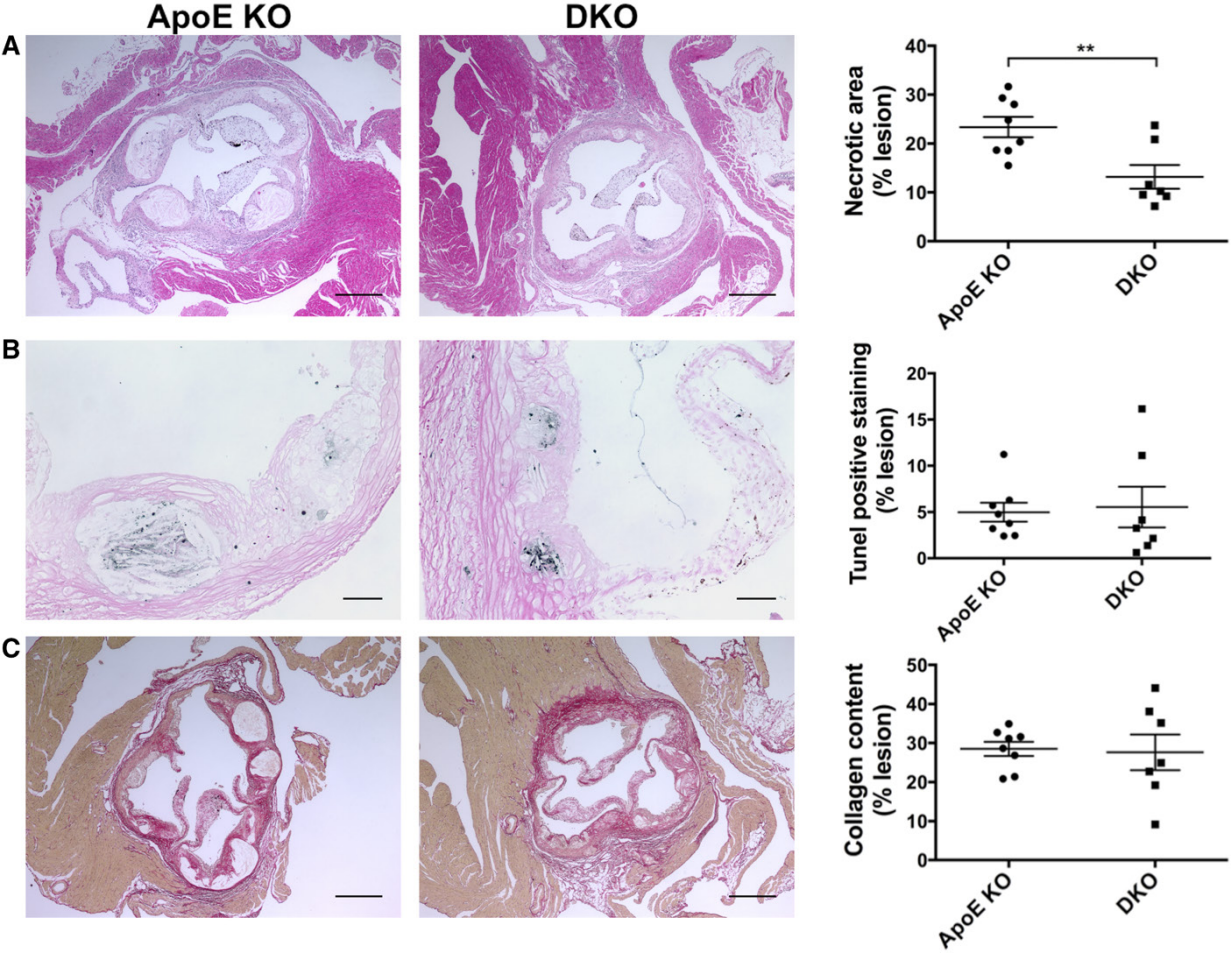
*Cd39* deletion results in less necrosis in advanced lesions from Western diet-fed mice. A: Acellular necrotic area was measured by hematoxylin and eosin staining on aortic sinus. B: Intra-lesion apoptosis was evaluated by TUNEL assay on aortic sinus. C: Intra-lesion collagen content was quantified by Sirius red staining on aortic sinus. Necrotic and collagen-rich fibrotic areas were measured in four 50 μm distant sections per each animal, while the TUNEL-positive area was measured in one section per animal (n = 8 *ApoE* KO and 7 DKO mice for all the analyses). Representative images are shown (scale bar is 250 μm in (A) and (C), 50 μm in (B). All quantification data are presented as mean ± SEM of the percentage staining of the total plaque area. Setting a color threshold in ImageJ identified positive staining. **P < 0.01.

We next investigated the effects of *Cd39* deletion on the composition of advanced lesions by analyzing the aortic sinus from Western diet-fed mice. Advanced lesions were mainly characterized by a necrotic core, which was surrounded by a collagen-rich fibrous cap (**Fig. 4**). Lesions in *ApoE* KO mice showed a necrotic area almost twice the size of that seen in DKO mice [23.4 ± 2.1% (n = 8) vs. 13.2 ± 2.4% (n = 7); *P* < 0.01; Fig. 4A]. This observation is not explained by the difference in apoptosis, as both groups had similar TUNEL-positive area [*ApoE* KO: 5.0 ± 1.0% (n = 8) vs. DKO: 5.5 ± 2.2% (n = 7); Fig. 4B]. Cleaved caspase 3 levels were very low in the macrophage-infiltrated areas in both groups (supplemental Fig. S3). No differences in the intra-lesion collagen content were revealed by Sirius red staining [*ApoE* KO: 28.5 ± 1.8% (n = 8) vs. DKO: 27.6 ± 4.6% (n = 7); Fig. 4C].

Taken together, these data show that the global deletion of *Cd39* results in decreased foam cell accumulation and impeded progression of atherosclerosis.

### *Cd39* deletion and platelet activation

**Fig. 5.**
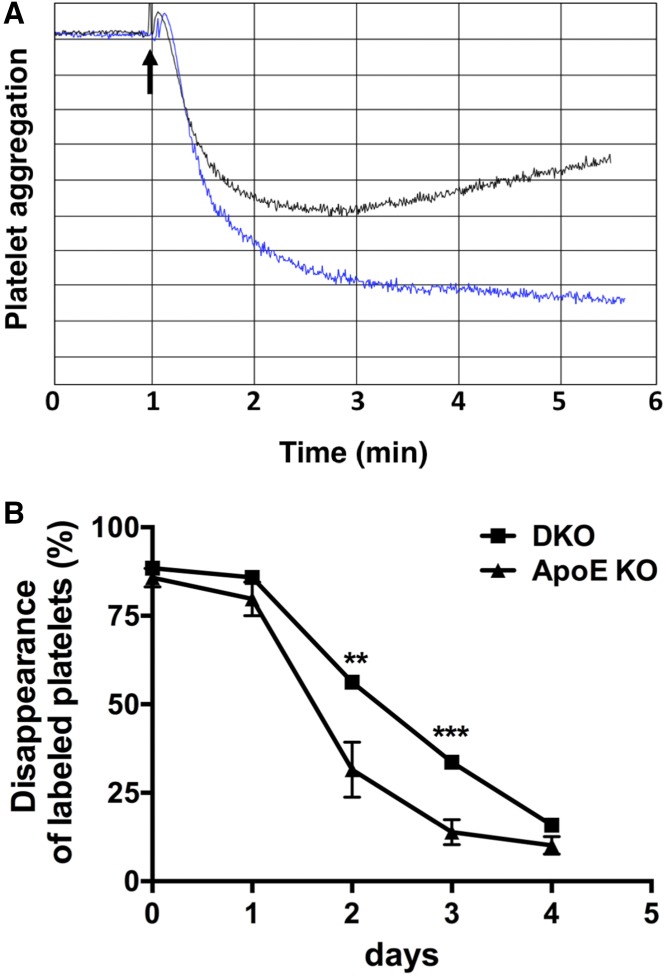
*Cd39* deletion impairs platelet activation. A: In vitro aggregometry response of platelets from *ApoE* KO and DKO mice fed the standard chow diet following 2.5 μM ADP stimulation. Data are presented as percent of light transmission over the time of incubation. Trace 1 (blue): platelets from *ApoE* KO mice; trace 2 (black): platelets from DKO mice. Aggregometry response is representative of three *ApoE* KO and four DKO mice. B: In vivo platelet turnover in *ApoE* KO and DKO mice fed the standard chow diet. Platelets were labeled and then counted every 24 h for 4 days, as described in the Materials and Methods. Data are presented as mean ± SEM of the percentage of labeled platelets to total platelets over the time of analysis (n = 5 *ApoE* KO and 4 DKO mice). **P < 0.01, ***P < 0.001.

Cd39 has an important role in the control of platelet activation and thromboregulation (14, 29). Therefore, we analyzed platelet function in *ApoE* KO and DKO mice. Platelets from DKO mice showed decreased in vitro aggregation, when compared with platelets from *ApoE* KO mice stimulated with the platelet agonist, ADP (**Fig. 5A**). The observed hyporeactivity is consistent with our prior reported desensitization of the P2y1 receptor in *Cd39*-null platelets (14).

Platelet hyporeactivity was confirmed in vivo in DKO mice (Fig. 5B). In fact, the platelet turnover was significantly higher in *ApoE* KO mice compared with DKO mice, as shown by the significant decrease of labeled platelet counts in *ApoE* KO mice at days 2 and 3 of analysis (Fig. 5B). These decreased turnover parameters in DKO mice suggest decreased platelet activation.

Taken together, these data show that the deletion of *Cd39* impairs platelet activation in hyperlipidemic mice.

### *Cd39* deletion increases macrophage cholesterol efflux

Macrophages play a central role in atherogenesis (3) and Cd39 regulates cell activation and myeloid responses to stimuli (13, 30). Hence, we next addressed whether *Cd39* deletion could impact lipid uptake by macrophages, foam cell viability, and cholesterol efflux.

**Fig. 6.**
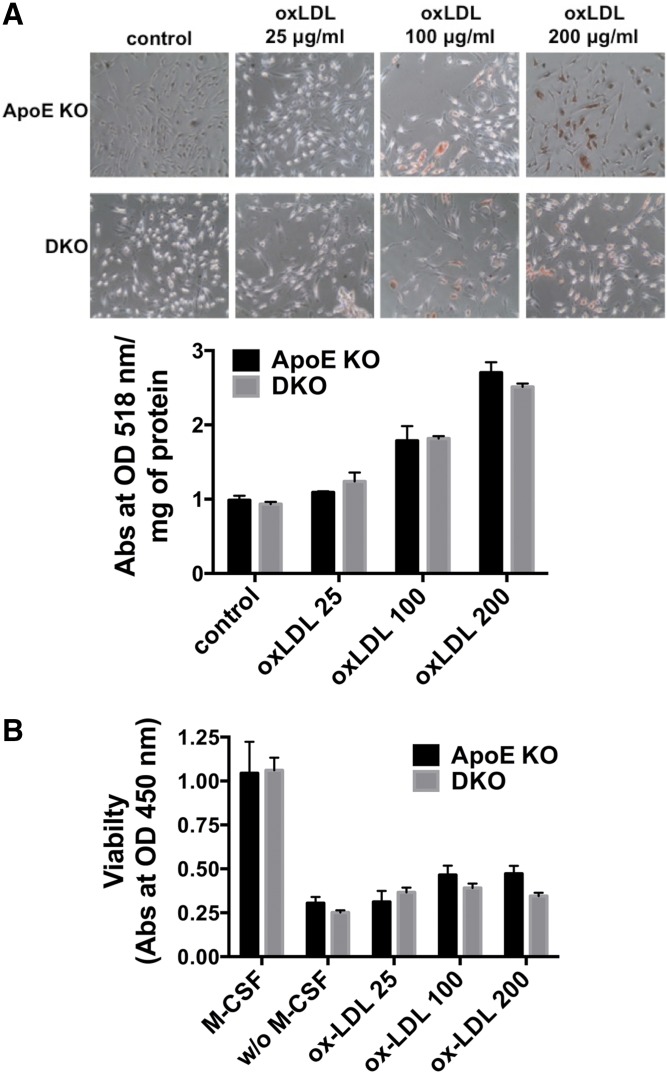
*Cd39* deficiency does not impact foam cell formation and viability of oxLDL-treated macrophages. A: *ApoE* KO and DKO BMDMs were incubated with 25, 100, or 200 μg/ml human oxLDL for 24 h in complete medium. Foam cell formation was evaluated by Oil Red O staining and Oil Red O quantification. Control: unstained cells. Representative images are shown (n = 3). B: *ApoE* KO and DKO BMDMs were incubated with 25, 100, or 200 μg/ml human oxLDL for 24 h in medium without M-csf. Viability was measured by Cell Counting Kit-8 assay. M-csf: control cells incubated in complete medium. w/o M-csf: control cells incubated in medium without M-csf (n = 4–8). All data are presented as mean ± SEM.

We first verified the differentiation of BMDMs and confirmed Cd39 expression by FACS (data not shown). We incubated *ApoE* KO and DKO BMDMs with 25, 100, and 200 μg/ml human oxLDL, and determined foam cell formation by Oil Red O staining as an indicator for lipid uptake. Both *ApoE* KO and DKO BMDMs became foam cells proportionally to the increasing concentrations of oxLDL (**Fig. 6A**).

Because macrophage survival and proliferation are predominant mechanisms of foam cell accumulation (31) and oxLDL can inhibit the apoptosis of macrophages (24, 25, 32, 33), we then tested the effect of *Cd39* deficiency in oxLDL-mediated survival of BMDMs. We treated BMDMs from *ApoE* KO and DKO mice with 25, 100, and 200 μg/ml human oxLDL in medium without M-csf (24, 25) for a period of 24 h and measured the macrophage viability. We noted that oxLDL induced slight increases of viability at higher oxLDL concentrations (100 and 200 μg/ml), as compared with M-csf withdrawal control (Fig. 6B). However, no significant differences in viability were detected between *ApoE* KO and DKO BMDMs at any tested concentrations of oxLDL (Fig. 6B).

Cholesterol efflux mediated by Abca1 is considered to be the main mechanism of cholesterol export from peripheral tissues and lipidation of HDL and other circulating apolipoproteins (34, 35). Recently, it has been shown that Abca1 increases extracellular ATP, a process that is required for cholesterol efflux to ApoA1 (20). We therefore tested whether *Cd39* deletion could impact cholesterol efflux.

**Fig. 7.**
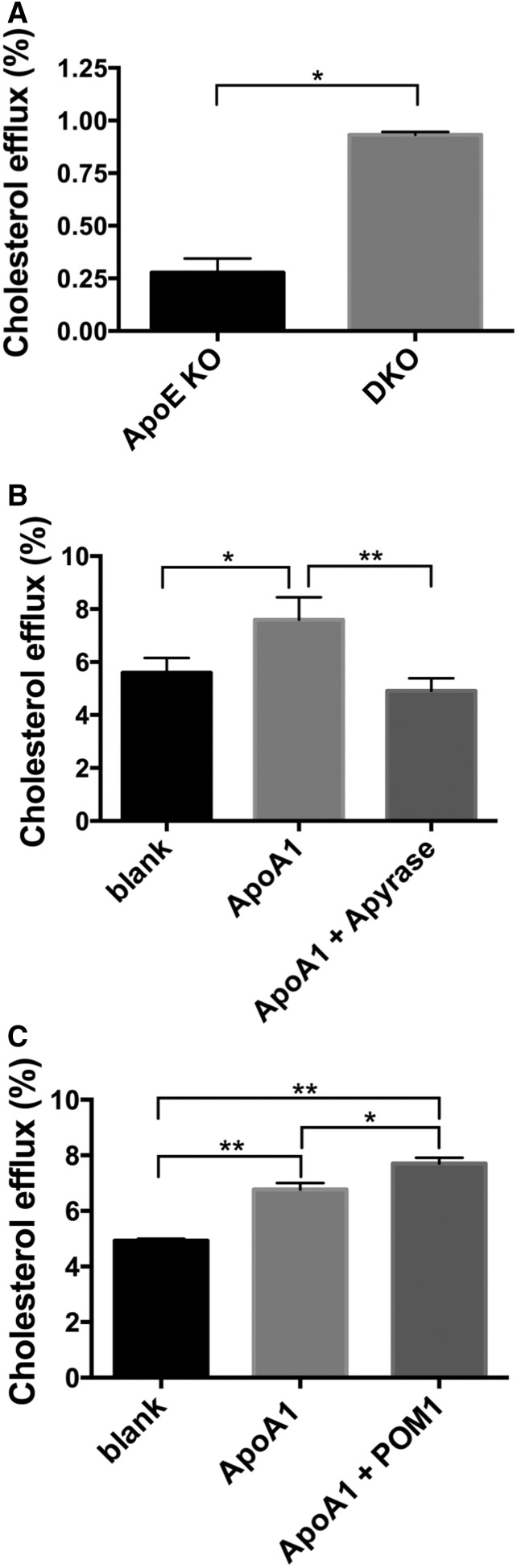
*Cd39* deletion results in increased macrophage Abca1-mediated cholesterol efflux. A: Cholesterol efflux to ApoA1 (10 μg/ml) was measured in *ApoE* KO and DKO BMDMs. Data are expressed as final efflux after subtracting the background (blank) to ApoA1 efflux (n = 2). B: Cholesterol efflux from DKO BMDMs was measured in the presence of ApoA1 or ApoA1 plus apyrase (25 U/ml) (n = 3). C: Cholesterol efflux from *ApoE* KO BMDMs was measured in the presence of ApoA1 or ApoA1 plus POM1 (26 μM) (n = 3). Blank: control with no acceptor (ApoA-1). All data are presented as mean ± SEM. *P < 0.05. **P < 0.01.

We found that the rate of Abca1-mediated cholesterol efflux to ApoA1 from macrophages of DKO mice was over 3-fold higher than that seen in macrophages of *ApoE* KO mice (**Fig. 7A**). The addition of apyrase reconstituted ATPase activity in DKO macrophages and dramatically decreased the cholesterol efflux to levels comparable to the background control (Fig. 7B). Similarly, the chemical inhibition of Cd39 by POM1 in *ApoE* KO macrophages significantly increased the rate of cholesterol efflux to ApoA1 (Fig. 7C).

Taken together, these data show that Cd39 regulates Abca1-mediated cholesterol efflux in macrophages by modulating the extracellular concentration of ATP. However, *Cd39* deficiency does not alter either the lipid uptake or the viability of macrophages following incubation with oxLDL in vitro.

### Deletion of *Cd39* in BM-derived cells exacerbates atherosclerosis

In order to determine the relative contribution of Cd39 between BM-derived and vascular tissue-resident cells, we performed BM transplantation experiments. Five-week-old *ApoE* KO and DKO mice were irradiated and underwent BM transplantation with either genotype, which gave rise to four experimental groups: *1*) wt → wt: *ApoE* KO recipient with *ApoE* KO BM donor; *2*) KO → wt: *ApoE* KO recipient with DKO BM donor; *3*) wt → KO: DKO recipient with *ApoE* KO BM donor; and *4*) KO → KO: DKO recipient with DKO BM donor. The successful engraftment of BM cells was confirmed by FACS analysis of Cd39/Cd19 cells from peripheral blood, as Cd39 is an established B-cell activation marker. As expected, Cd39/Cd19 double-positive cells were present at similar levels in wt → wt and wt → KO recipients, whereas these cells could not be found in KO → wt or KO → KO recipients (supplemental Fig. S4A).

Moreover, CD39 staining in the aortic sinus from wt → wt recipients indicated the expression of Cd39 not only in resident cells, such as endothelial cells and VSMCs, but also in foam cells, which are known to be derived mostly from circulating monocytes (36). Cd39 expression was mainly detected in endothelium and VSMCs in KO → wt recipients, but only in foam cells in wt → KO recipients (supplemental Fig. S4B).

**Fig. 8.**
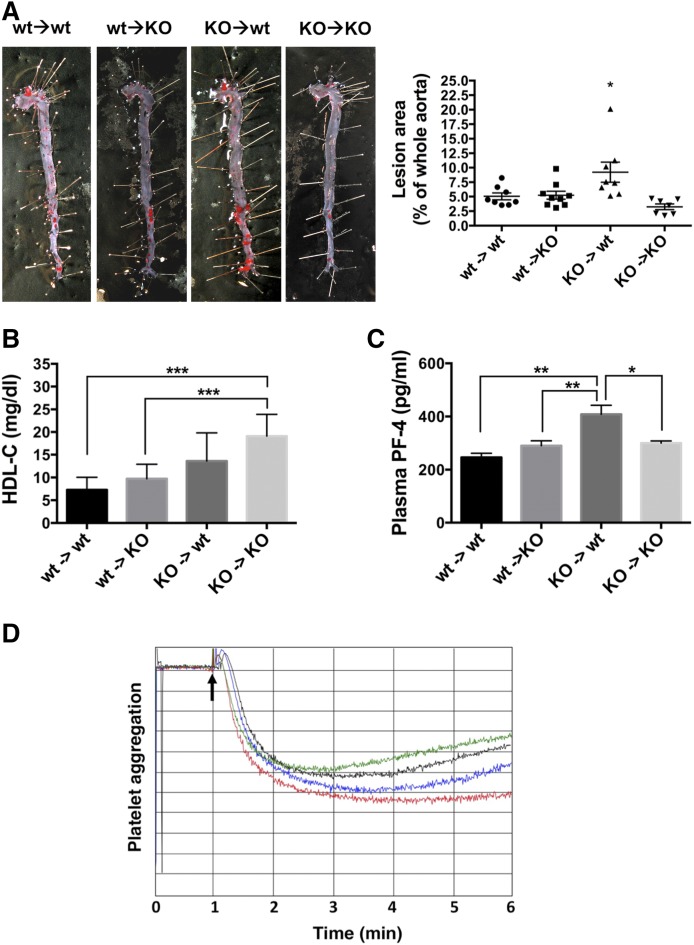
*Cd39* deletion in BM-derived cells exacerbates atherosclerosis and provokes platelet activation. Recipient *ApoE* KO and DKO mice were lethally irradiated and then transplanted with BM cells from either *ApoE* KO (wt) or DKO (KO) donors. Mice were fed a Western diet for 12 weeks. A: Atherosclerosis lesion size was evaluated by Oil Red O staining of the whole aorta (n = 8 wt → wt, 9 wt → KO, 8 KO → wt, and 7 KO → KO mice). Representative images are shown. B: HDL-C concentration was measured in plasma from transplanted mice (n ≥ 5 mice per group). C: Pf4 concentration in plasma from transplanted mice was determined by ELISA (n = 7 wt → wt, 9 wt → KO, 7 KO → wt, and 5 KO → KO mice). All data are presented as mean ± SEM. *P < 0.05, **P < 0.01, ***P < 0.001. D: In vitro aggregometry response of platelets from BM-transplanted mice fed a standard Chow diet following 2.5 μM ADP stimulation. Data are presented as percent of light transmission over the time of incubation. Trace 1 (blue), platelets from wt → wt mice; trace 2 (black), platelets from wt → KO mice; trace 3 (red), platelets from KO → wt mice; and trace 4 (green), platelets from DKO mice. Aggregometry responses are representative of three mice per group.

We stained the entire aorta with Oil Red O dye to evaluate the progression of atherosclerosis. Surprisingly, KO → wt mice had exacerbated atherosclerosis with significantly larger area of lesions than those in other groups [9.2 ± 1.7% (n = 8); *P* < 0.05; **Fig. 8A**]. The wt → KO and wt → wt mice showed similar size of lesions of 5.2 ± 0.7% (n = 9) and 5.1 ± 0.6% (n = 8), respectively (Fig. 5A). The KO → KO group had the smallest lesion sizes, consistently, within the analysis in nontransplanted mice (Fig. 1) [3.2 ± 0.5% (n = 7); Fig. 8A]. Moreover, this group also showed significantly higher plasma HDL-C levels than wt → wt and wt → KO mice (*P* < 0.001; Fig. 8B).

Hemizygous deletion of *Cd39* (*Cd39*^+/−^) on *ApoE* KO atherosclerotic background results in heightened platelet activation and consequent worsening of atherosclerosis (19). Therefore, we analyzed Pf4, a chemokine released during platelet activation (37), in the plasma from transplanted mice. The KO → wt mice had significantly higher plasma concentrations of Pf4 over all other groups (Fig. 8C). We further measured the in vitro aggregation of platelets from BM-transplanted mice (Fig. 8D). In agreement with the highest levels of plasma Pf4, platelets from KO → wt mice exhibited the greatest aggregation in response to ADP stimulation, when compared with the other groups (Fig. 8D).

Taken together, these data show that the deletion of *Cd39* in BM-derived cells alone exacerbated atherosclerosis. This phenotype also appears to be mediated, at least in part, by heightened levels of platelet activation, as previously described (19). Furthermore, the global deletion of *Cd39* is required to provide protection against development of atherosclerosis.

### *Cd39* deletion does not impact *ApoE*-null VSMCs migration and proliferation in vitro

Because proliferation and migration of VSMCs occur during atherogenesis (1, 4), we isolated VSMCs from the aorta of *ApoE* KO and DKO mice and performed in vitro proliferation and migration assays.

VSMCs proliferated strongly in response to 10% FBS. A slight increase in proliferation was noted in response to Pdgf-β, whereas extracellular ATP did not alter VSMC proliferation. However, no differences were detected between the two groups in each condition tested (supplemental Fig. S5B). No differences in the number of migrated cells were found between *ApoE* KO and DKO VSMCs (supplemental Fig. S5C).

These data show that global *Cd39* deficiency does not overtly impair the migration or proliferation of VSMCs obtained from hyperlipidemic mice.

## DISCUSSION

Because of the largely protective roles afforded by Cd39 in inflammatory disorders, thrombosis, and tissue ischemia (14, 16, 38), we hypothesized that the total lack of Cd39 might result in a severe atherosclerotic phenotype in *ApoE*-deficient mice. Paradoxically, we found that the total deletion of *Cd39* was protective against atherosclerosis. This observation was even more curious, given that *Cd39*-null mice are insulin resistant, mildly hypertensive, exhibit a pro-inflammatory phenotype (14, 39), and have evidence for macrophage-monocyte activation (30).

The DKO mice exhibited significant decreases in size of atherosclerotic lesions when compared with the *ApoE* KO mice, following both chow and high-fat diet feeding (Fig. 1). DKO mice had higher serum levels of HDL following Western diet feeding (Fig. 2). The fatty streaks in DKO mice had decreases in numbers of macrophages (Fig. 3A). Lesions of *ApoE* KO mice also showed a trend toward increased TUNEL staining, suggesting a more advanced formation of necrotic cores, compared with DKO mice (Fig. 3C). Consistently, the atherosclerotic plaques of DKO mice fed with Western diet had necrotic cores around half the size of those seen in *ApoE* KO mice (Fig. 4A), further suggesting less disease progression. However, deletion of *Cd39* in BM-derived cells alone exacerbated atherosclerosis in chimeric mice (Fig. 8). These data suggest a complex role of Cd39 in this chronic multifaceted inflammatory disease model.

Platelets play a pivotal role in atherosclerosis, contributing to both the onset and progression of disease (40). Activation of platelets following endothelium injury and interaction with oxLDL and endothelial cells release pro-inflammatory agonists that cause further inflammation and leukocyte recruitment (40). It has been shown that the injection of activated platelets in *ApoE* KO mice promotes leukocyte binding to endothelium and higher inflammation leading to exacerbation of atherosclerosis (41). Furthermore, activated platelets promote oxidative modification of LDL contributing to the accumulation of oxLDL, which in turn amplify platelet activation (42, 43). On the contrary, platelet hyporeactivity provides protection against atherosclerosis development (37, 44) and administration of, for example, aspirin and clopidogrel decreases platelet thrombus formation following rupture of atherosclerotic plaque in vivo (45).

Here, DKO mice show decreased in vitro platelet aggregation following ADP stimulation (Fig. 5A), as well as decreased in vivo platelet turnover, when compared with *ApoE* KO mice (Fig. 5B). These data are in agreement with the reported platelet hyporeactivity phenotype described in normolipidemic mice with global deletion of *Cd39* (14). Similarly, atherosclerotic mice deficient for the ADP receptors, *P2y1* or *P2y12*, show decreased platelet activation, likewise, along with the atheroprotective phenotype (37, 44). Given the well-known role of platelets in atherosclerosis, we believe that the observed platelet hyporeactivity in DKO mice may provide a substantial mechanism of protection against the development of disease.

In a very interesting report, Kanthi et al. (19) have shown that the total deletion of *Cd39* in atherosclerotic mice did not result in protection against atherosclerosis, despite these mice clearly showing impaired platelet functions. In that study, DKO mice did not exhibit a significantly different atherosclerotic phenotype as compared with the other groups. However, the overall phenotypic distribution was heterogeneous with a seemingly bimodal distribution of atheromatous disease in the fully null *Cd39*^−/−^ mice (19). Potential reasons for this difference with our data may be that the *Cd39* KO mice described by Kanthi et al. (19) derived from mice that were generated by a different KO strategy and/or housed in a different facility (46). Furthermore, in this recent study the authors used a slightly different high-fat diet to what we used (47).

It has been demonstrated that platelet hyporeactivity in global *Cd39*-null mice is a consequence of P2y1 receptor desensitization (14, 19). In agreement with the atheroprotective phenotype observed in our DKO mice, the genetic inactivation of *P2y1* in *ApoE* KO mice also results in platelet hyporeactivity and protection from atherosclerosis (44). However, these authors have shown that vascular nonhematopoietic-derived cells expressing *P2y1* receptors play an important role in disease evolution (44). Interestingly, *P2Y1/ApoE* DKO mice show a trend toward decreased plasma cholesterol levels, suggesting a role of *P2y1* in lipid metabolism (44).

Overall, these findings suggest that platelet desensitization may partially explain the observed atheroprotective phenotype in DKO mice, but that other factors may also be operative.

Macrophages play a central role in atherosclerosis by scavenging excessive cholesterol and modified LDL, therefore resulting in foam cell development in the nascent plaque (1). Dysregulated intracellular lipid trafficking, metabolism, and efflux lead to foam cell death, which promotes the formation of necrotic core and the progression of atherosclerosis (48). Changes in macrophage pathophysiology in DKO mice may explain the smaller plaques, diminished macrophage content, and smaller necrotic area than in *ApoE* KO mice (Figs. 1, 3, 4).

To test such a hypothesis, we addressed the effects of *Cd39* deletion on three aspects of macrophage physiology: oxLDL-induced foam cell formation, foam cell viability, and cholesterol efflux to ApoA1. Following incubation with oxLDL, we observed no differences between foam cell formation in *ApoE* KO and DKO BMDMs (Fig. 6A). This result is consistent with previous evidence suggesting the lack of difference in Dil-oxLDL uptake between peritoneal macrophages from DKO and *ApoE* KO mice (19).

Macrophage survival and proliferation, which are promoted by oxLDL in atherosclerotic lesions, have recently been recognized as one of the dominant mechanisms of foam cell accumulation during atherogenesis (31, 32). Still, we did not detect differences in foam cell viability between *ApoE* KO and DKO BMDMs at any concentrations of oxLDL tested (Fig. 6B). There was also no difference in the level of foam cell apoptosis between *ApoE* KO and DKO mice by TUNEL and cleaved caspase 3 staining (Fig. 4B, supplemental Fig. S3). These findings are unlike those seen in hyperlipidemic *A2a*-null mice, where lack of adenosine signaling compromises anti-apoptotic pathways in macrophages resulting in depletion of foam cells in atherosclerotic lesions (49). Taken together, our data suggest that the absence of *Cd39* does not impact foam cell formation or macrophage survival.

It has been shown that the lipid transporter Abca1 increases extracellular ATP, which, in turn, facilitates cholesterol efflux to ApoA1 (20). The Abca1-mediated cholesterol efflux process appears to be impacted by the extracellular ATP concentration per se, rather than by the modulated ATP signaling through purinergic receptors (20). Cd39 is the dominant macrophage ectonucleotidase, which regulates pericellular concentrations of ATP (13). In agreement with such data, we observed increased levels of cholesterol efflux from DKO BMDMs (Fig. 7A). We also noted that alteration of extracellular ATP levels regulated this process (Fig. 7B, C).

Cholesterol efflux data are consistent with increased serum HDL-C levels in DKO mice (Fig. 2C). HDL maturation is mainly achieved by the lipidation of nascent apolipoproteins, mostly ApoA1 through cholesterol efflux from peripheral tissues following interaction with Abca1 transporters (34, 35). The capacity of cholesterol efflux from macrophages is inversely associated with atherosclerosis (50) and, accordingly, our DKO mice show less development of atherosclerosis. We cannot exclude salutary effects of *Cd39* gene deletion on the cholesterol efflux from peripheral tissues. However, our data suggest that the enhanced cholesterol efflux and serum HDL levels in DKO mice might contribute to the atheroprotective phenotype observed in DKO mice; along with the acquired, impaired platelet functionality noted.

Given the observed enhancement in cholesterol efflux in DKO BMDMs, we have expected that KO → wt BM transplants in chimeric mice might have afforded protection against the development of atherosclerosis. Curiously, this group exhibited some exacerbation in disease (Fig. 8). This unexpected result might be explained by the observed platelet hyperreactivity in these mice, given that the endothelium expresses Cd39 (Fig. 8C).

Extracellular ADP is a potent platelet activator that induces the release of chemokines, such as Pf4 (and Rantes), from α granules, as noted here, by signaling through P2y receptors (37). It has been shown that in partial *Cd39* deficiency, platelets show increased in vitro ADP-induced activation that is consistent with augmented levels of platelet-secreted chemokines and exacerbated atherosclerosis in vivo [see (19)]. In agreement with such findings, we observed higher in vitro aggregation in platelets from KO → wt mice following ADP stimulation (Fig. 8D). Furthermore, KO → wt mice showed higher levels of circulating Pf4 when compared with the other groups (Fig. 8C). Consistently, these mice exhibited exacerbations in the extent of atherosclerosis (Fig. 8A).

Concordant with the global *Cd39*-null mouse phenotype (Fig. 1), KO → KO mice also show decreased atherosclerosis (Fig. 8). Moreover, these chimeric mice also show higher levels of protective HDL-C, when compared with the other groups (Fig. 8B). Taken together, these findings confirm that Cd39 serves as vascular thromboregulatory factor, differentially impacting platelet reactivity and cholesterol metabolism in partial or global deficient animals, in a manner seemingly dependent upon extracellular nucleotide concentrations.

We have confirmed high-levels of expression of Cd39 in VSMCs (supplemental Fig. S1A), another cellular contributor in atherogenesis (1). During the early stage of plaque development, media-derived VSMCs migrate into the intima following a gradient of chemokines and growth factors, in particular Pdgf-β (1, 4). These VSMCs proliferate and produce extracellular matrix, participating in the formation of the fibrous cap (1). Somewhat different to previous data on *Cd39*-null VSMCs obtained from normolipidemic mice (51), we did not observe significant differences in the rate of cell migration or proliferation following stimulation with Pdgf-β, FBS, and ATP (supplemental Fig. S5).

In summary, we have shown that the global deletion of *Cd39* is atheroprotective in *ApoE* KO mice. The atheroprotective phenotype observed in DKO mice is associated with platelet hyporeactivity, increased cholesterol efflux from macrophages, and higher serum levels of HDL-C. These findings indicate the complexity of purinergic signaling in atherosclerosis.

## Supplementary Material

Supplemental Data
