# Supplementary material for: Complete deletion of Cd39 is atheroprotective in apolipoprotein E-deficient mice
Source: J Lipid Res. 2017 May 9;58(7):1292–305. doi: 10.1194/jlr.M072132 (PMC5496028; doi:10.1194/jlr.M072132)
Supplement: Supplemental Data [file supp_58_7_1292__index.html]

Complete deletion of Cd39 is atheroprotective in apolipoprotein E-deficient mice — Complete deletion of Cd39 is atheroprotective in apolipoprotein E-deficient mice — Supplemental Data 

# Complete deletion of *Cd39* is atheroprotective in apolipoprotein E-deficient mice

## Supplemental Data

- Supplemental Material (.pdf, 3.0 MB) - Supplemental Material
