# Supplementary material for: Complete deletion of Cd39 is atheroprotective in apolipoprotein E-deficient mice
Source: J Lipid Res. 2017 May 9;58(7):1292–305. doi: 10.1194/jlr.M072132 (PMC5496028; doi:10.1194/jlr.M072132)
Supplement: Supplemental Data [file 10.1194_M072132_jlr.M072132-1.pdf]

SUPPLEMENTAL MATERIAL

Complete deletion of Cd39 is atheroprotective in apolipoprotein E-deficient mice

Marco De Giorgi[1], Keiichi Enjyoji[1], Gordon Jiang[1], Eva Csizmadia[1], Shuji Mitsuhashi[1], Richard J. Gumina[2], Ryszard T. Smolenski[3], and Simon C. Robson[1]

[1] Transplant Institute and Hepatology, Department of Medicine, Beth Israel Deaconess Medical Center and Harvard Medical School, Boston, MA, USA.

[2] Department of Medicine, Vanderbilt University Medical Center, Nashville, TN, USA.

[3] Department of Biochemistry, Medical University of Gdansk, Gdansk, Poland.

SUPPLEMENTAL FIGURES AND FIGURE LEGENDS

Supplemental Figure S1

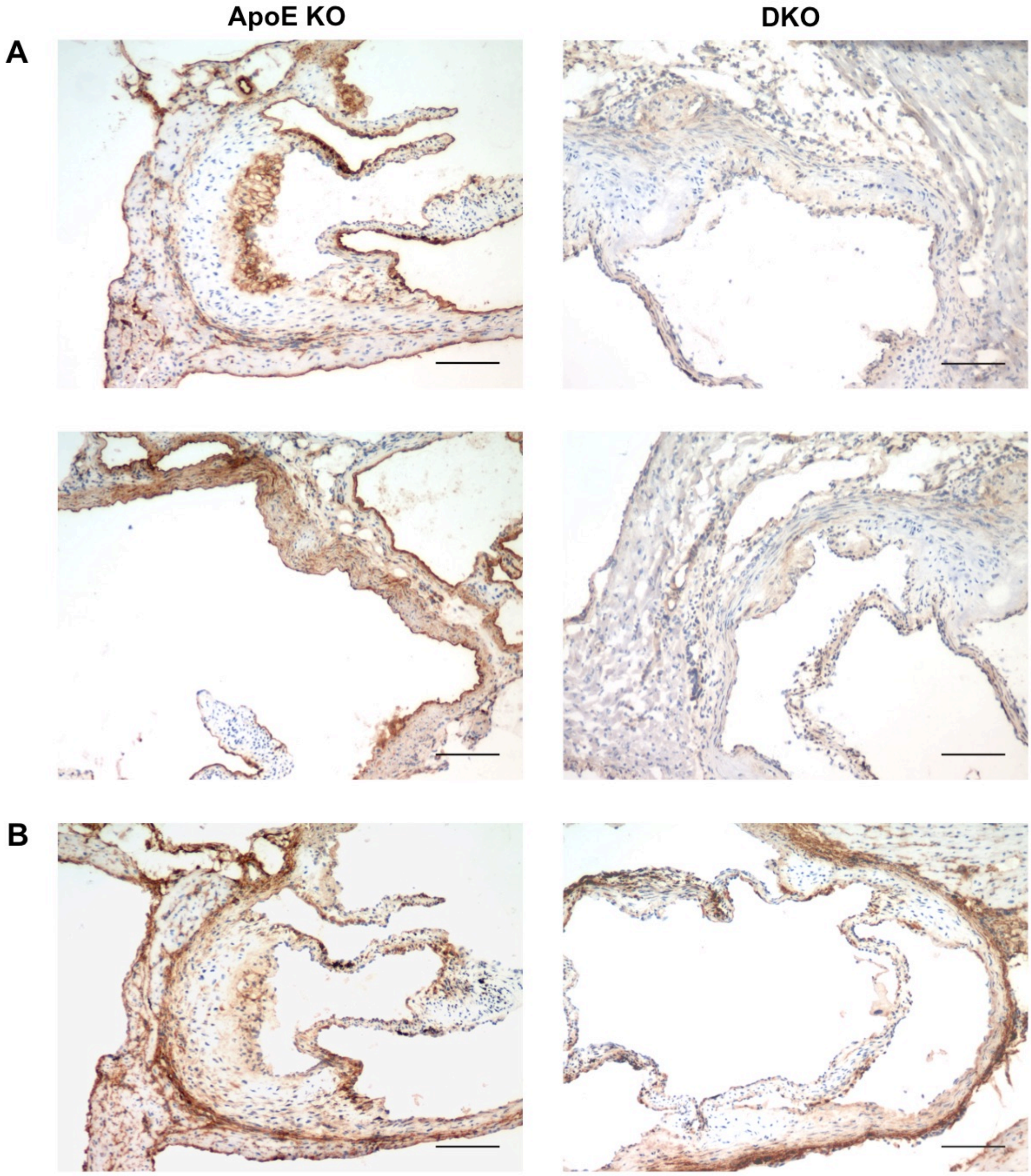

Supplemental Figure S1. Major ectonucleotidases in the aortic sinus of atherosclerotic mice. Immunohistochemical staining of CD39 (**A**) and CD39L1 (**B**) in the aortic sinus of chow diet-fed ApoE KO and DKO mice. Scale bar is 100 μm.

Supplemental Figure S2

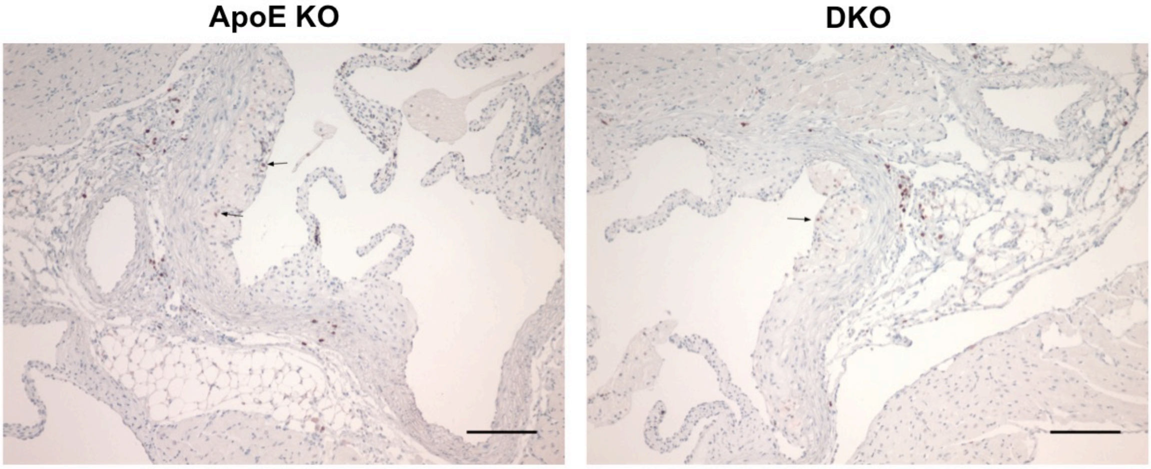

Supplemental Figure S2. Immunohistochemical staining of T-cells in aortic sinus from chow diet-fed ApoE KO and DKO mice. Arrows indicate intra-lesion CD3 positive cells. Representative images are shown. Scale bar is 100 μm.

Supplemental Figure S3

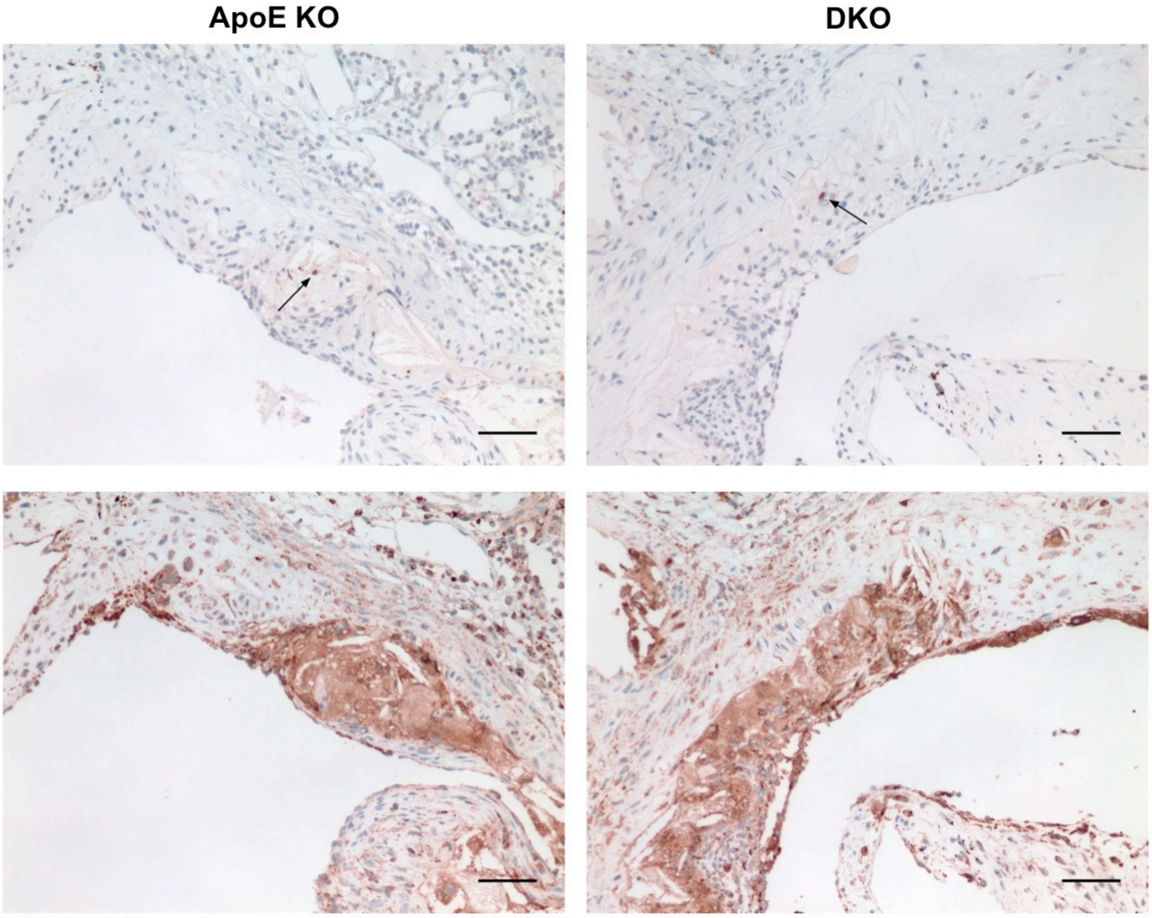

Supplemental Figure S3. Immunohistochemical staining of cleaved caspase 3 in aortic sinus from Western diet-fed ApoE KO and DKO mice. Arrows indicate cleaved caspase 3 positive cells. MAC3 staining of a 5 μm distant section indicates that the cleaved caspase 3 positive cells are macrophages. Representative images are shown. Scale bar is 50 μm.

Supplemental Figure S4

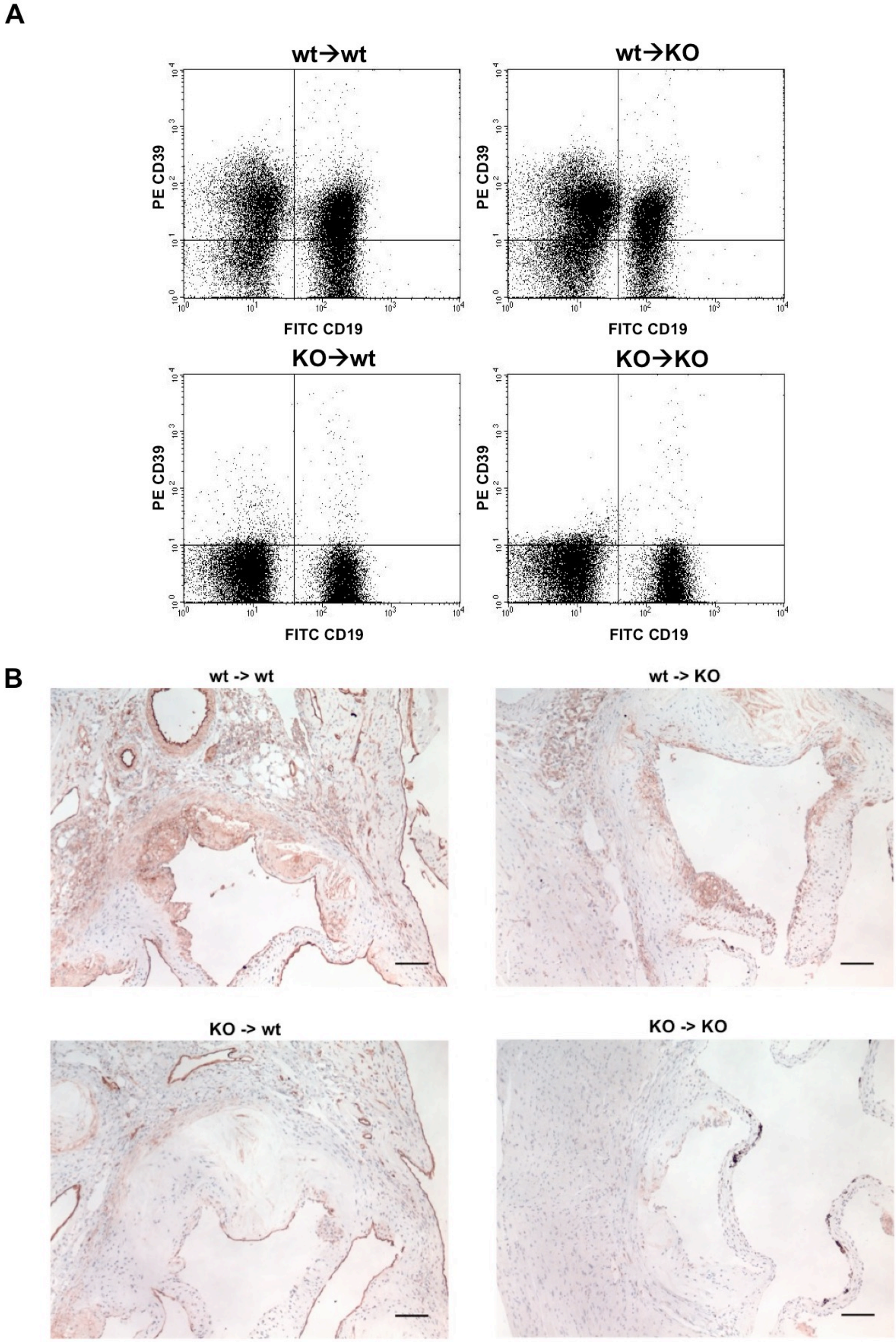

Supplemental Figure S4. Successful BM reconstitution in transplanted mice. (**A**) FACS analysis of CD39 on CD19 positive B cells from peripheral blood of transplanted mice. A representative dot blot is shown per each group. (**B**) CD39 immunohistochemical staining of aortic sinus from BM transplanted mice. A representative image is shown per each group. Scale bar is 100 μm.

Supplemental Figure S5

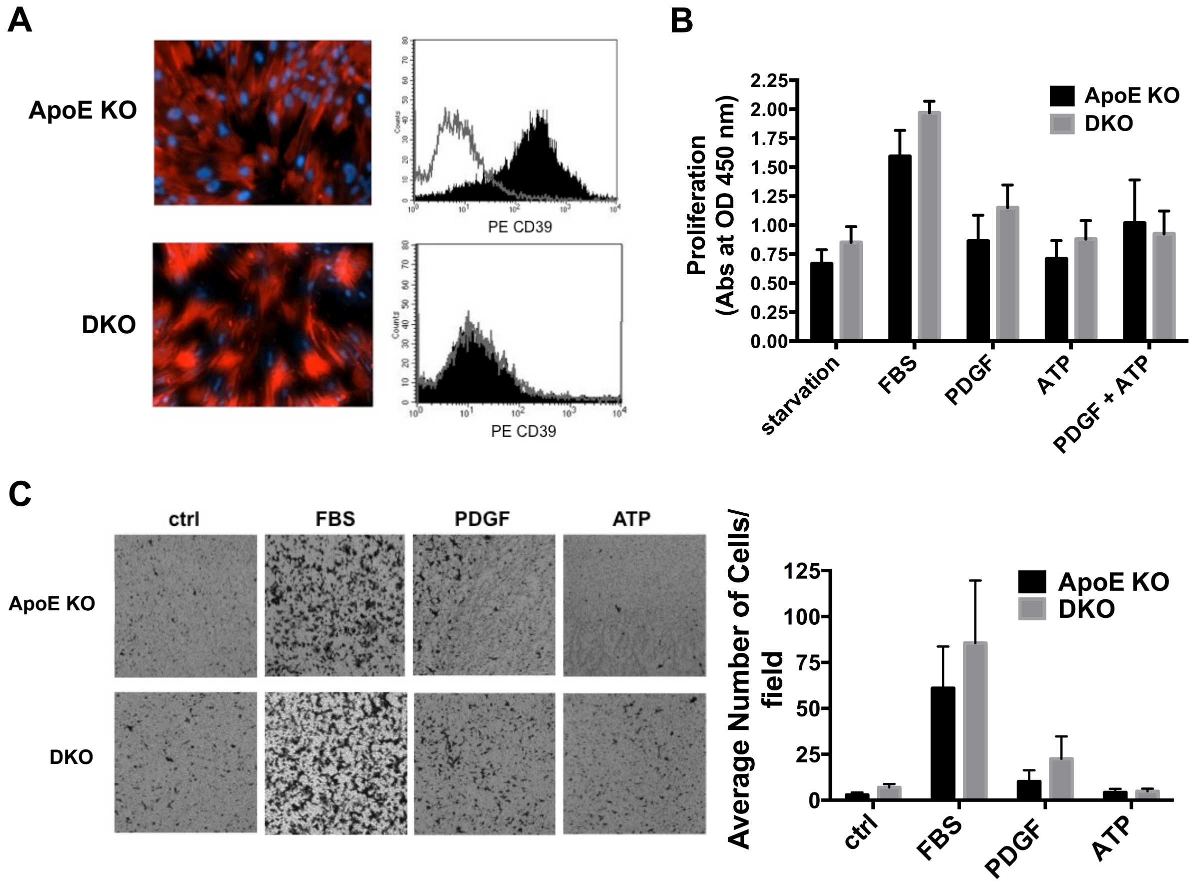

Supplemental Figure S5. Intact *in vitro* VSMC proliferation and migration in global CD39 deficiency. (**A**) VSMC purity and CD39 expression were evaluated by SMC alpha actin immunofluorescence and FACS analysis respectively. (**B**) VSMC proliferation in response to 10% FBS, 10 ng/ml PDGF, 100 μM ATP or PDGF plus ATP combination was evaluated after 48 hours by CCK8 assay (n=4-5). (**C**) VSMC migration towards 10% FBS, 10 ng/ml PDGF or 100 μM ATP was investigated using a Transwell system (n=3). All data are presented as mean ± SEM.
